# The importance of the auditory evoked potential in acoustic neuromas

**DOI:** 10.1016/S1808-8694(15)30620-0

**Published:** 2015-10-18

**Authors:** Ana Helena Bannwart Dell’Aringa, Luiz Fernando Pires Sena, Rodrigo Teixeira, Alfredo Rafael Dell’Aringa, José Carlos Nardi

**Affiliations:** 1 Specialized speech therapist, Otorhinolaryngology Discipline, Faculdade de Medicina de Marilia.; 2 Physician, resident in Otorhinolaryngology.; 3 Physician, resident in Otorhinolaryngology.; 4 Doctor in otorhinolaryngology. Head of the Otorhinolaryngology Discipline, Faculdade de Medicina de Marilia.; 5 Master's degree in otorhinolaryngology, Faculty of the Faculdade de Medicina de Marília.

**Keywords:** hearing loss, acoustic neuroma

## INTRODUCTION

Hearing loss, frequently with tinnitus, is the main symptom of acoustic neuromas (ANs), which compress the cochlear nerve and affect the cochlear blood supply. This vascular mechanism explains why atypical, fluctuating deafness, with audiometric features of retrocochlear involvement, may occur in this condition.^1^

An early diagnosis of ANs is essential for a good prognosis. Physicians should recognize the clinical signs of neurinomas, particularly in individuals aged just over 40 years; this is true even in cases of symmetrical sensorineural hearing loss.

The brainstem auditory evoked potential (BAEP) is an objective, non-invasive method for a neurophysiological analysis of auditory pathways from the inner ear to the high brainstem. It is a short latency potential that generates a wave series (from I to VII) that appears within the first 10 ms after a sound stimulus is presented. These waves are generated due to the sequential activation of auditory pathway structures, and are picked up by electrodes placed on the skin.^2^

## CASE REPORT

**Figure 1:**
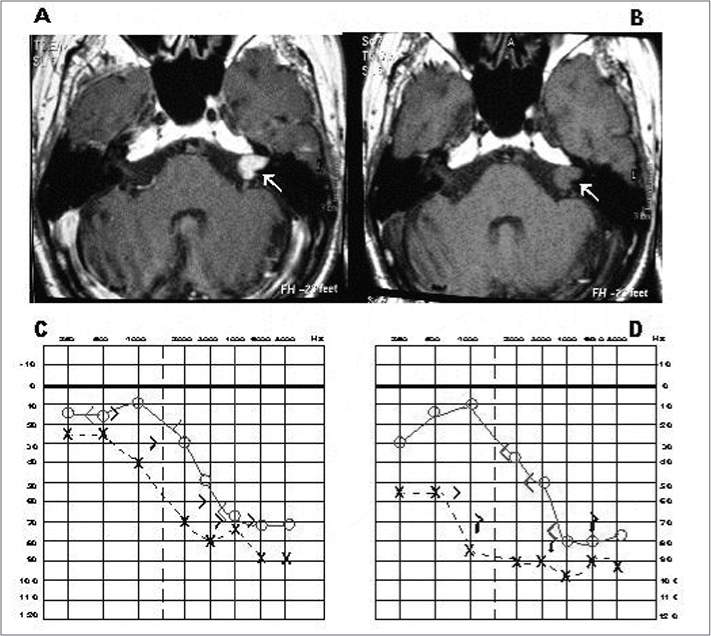
MRI images with (A) and with no contrast media (B), and audiometries done before (C) and after confirming the AN (D).

A male Brazilian patient aged 74 years, born in Itatiba, was referred to the ENT outpatient unit from another town for the hearing aid program. He complained of decreased hearing in the left ear. The patient complained of dysacusis and ear fullness in the left ear for the past 2 years; there was no dizziness or tinnitus. Pure tone audiometry showed mild downward sloping sensorineural dysacusis in the left ear, and decreased hearing over 2 KHz in the right ear (Figure 1). The speech recognition index was 88% at 85 dB in the left ear, and 96% at 60 dB in the right ear. The BAEP revealed an increased wave V latency time and an interpeak interval I-V in the left ear; OAE-DP were absent from 1,031 to 6,703 HZ in the left ear. Magnetic resonance imaging (MRI) was done on 08 May 2006, showing an expanding intracanalicular lesion to the left hat extended partially to the pontocerebellar cistern measuring 15 x 12 mm, which suggested an AN (Figure 1).

A second audiometry one year later revealed severe flat sensorineural dysacusis in the left ear, and mild sloping sensorineural hearing loss in the right ear (Figure 1); the speech recognition index was 10% at 85 dB (left ear) and 100% at 35 dB (right ear).

## DISCUSSION

During the 1980s, MRI started to be used in the diagnosis of small lesions (its sensitivity for small tumors is 100%^3^), which increased the possibility of an early diagnosis and surgery of AN.

According to some authors, the sensitivity of BAEP varies with the tumor size; it is, therefore, a less reliable method. Dornhoffer et al. reported a 93% sensitivity for tumors measuring less than 1 cm.^4^ Schmidt et al. (2001) reported a 58% sensitivity for ANs measuring less than 1 cm, a 94% sensitivity for ANs between 1.1 and 1.5 cm, and a 100% sensitivity for tumors over 1.5 cm; the general sensitivity was 90%.^5^

The advantage of BAEP is its lower cost; it may be done first to reduce the number of patients requiring MRI. If retrocochlear disease is suspected, MRI may be done even if the BAEP is normal, given its sensitivity range according to the tumor size.

## FINAL COMMENTS

This paper aimed to demonstrate the need and importance of a complete audiological evaluation in all cases of bilateral sensorineural dysacusis with minor asymmetry.
